# Editorial: Plant bioactives: challenges of extraction and processing

**DOI:** 10.3389/fnut.2024.1357925

**Published:** 2024-01-15

**Authors:** Allah Rakha, Aamir Shehzad, Kamran Khan

**Affiliations:** ^1^National Institute of Food Science and Technology, University of Agriculture, Faisalabad, Pakistan; ^2^UniLaSalle, Univ. Artois, ULR7519 - Transformations & Agro-ressources, Normandie Université, Mont-Saint-Aignan, France; ^3^Department of Food Science, GC University, Faisalabad, Pakistan

**Keywords:** plant bioactives, nutraceuticals, extraction techniques, food application, health benefits

Plant bioactives are the secondary metabolites that induce physiological responses in the human body. These compounds have demonstrated pharmacological effects in both humans and animals (1, 2). Moreover, they exhibit antioxidant, anticarcinogenic, anti-allergic, antimutagenic, anti-inflammatory, and antimicrobial properties among others (3). Purposely, plant bioactives hold significance in the food, pharmaceutical, and chemical sectors.

Natural bioactive compounds can be categorized into various groups including polyphenols, phytosterols, terpenoids, carotenoids, polysaccharides, capsaicinoids, saponins, alkaloids, glucosinolates, tocopherols, and other compounds (4). They are present in various fruits, vegetables, and herbs, as well as are extracted from agro-industrial residues, fungi, animals, and bacteria (1, 5). The incorporation of these bioactives in foods and other value-added products necessitates the application of sustainable extraction techniques. Several techniques have been utilized to optimize extraction conditions for phenolics and associated compounds (6).

The process of extraction serves as a primary step in the isolation and purification of bioactives from different food sources. Some phenolic acids are water soluble and can be extracted with ease while insoluble secondary metabolites present a greater challenge. Although, maceration, Soxhlet, and heat reflux are all viable methods for extracting bioactive compounds, however, their operational parameters greatly differ. The technological processes for the extraction of bioactive compounds from foods should prioritize the attainment of exceptional quality products while simultaneously ensuring sustainability, efficiency, and cost-effectiveness. Furthermore, due to the increasing demand of consumers for chemical-free products and industrial concerns regarding sustainable extraction processes, extensive research has been conducted by the food sector to develop new and advanced extraction technologies. Purposely, cutting-edge technologies such as pulse electric field, high hydrostatic pressure, ultrasonic, and supercritical fluid are rapidly replacing traditional methods. Hence, the utilization of innovative and novel technologies may have the potential to achieve higher yields and extraction rates. Besides, these may employ a variety of inorganic solvents, require reduced energy consumption, and effectively preserve thermosensitive chemicals in the final extract (7).

This Research Topic “*Plant bioactives: challenges of extraction and processing*” was designed to gather articles that are appropriate for enhancing our understanding and knowledge concerning plant bio-actives. It also aims to explore the optimization of bioactive compound extraction parameters and their application within the food industry. Furthermore, it seeks to investigate the impact of processing conditions on the bioavailability and functional properties of these bio-actives. Lastly, this Research Topic intends to examine emerging technologies that can be utilized to preserve and enhance the bioactivities of nutraceuticals.

In this particular Research Topic, four articles are published. One of them is an original research article on the optimization of ultrasonic-assisted extraction parameters for flavonoids and their antioxidant properties. The second article is a mini-review that describes the functional, nutritional properties, and bioavailability of garlic due to processing. The remaining contributions include two review articles about the emerging technologies for preserving bioactivity and enhancing nutraceutical applications of bio-flavonoid-rich plant sources and applications of bioactive compounds-rich extracts in different food products. Regarding the original research article, the optimization of ultrasound-assisted extraction (UAE) to enhance the yield and the antioxidant properties of flavonoids from bitter *Lactuca indica L. cv* leaves was studied. The results showed that the ultrasound-assisted method had the best extraction capacity compared to solvent and microwave-assisted extraction in terms of yields and antioxidant activity. Furthermore, using an integrated metabolic approach involving ultra-high performance liquid chromatography-mass spectrometry quantification, the authors were able to show a significant change in the flavonoid concentration of flowers, leaves, stem, and roots since flowers exhibited the highest concentration of flavonoids. Overall, the study presented is of high relevance for the ultrasound-assisted method to offer an efficient and green technique for the extraction of flavonoids from *Lactuca indica L. cv*, with potential applications in the pharmaceutical and food industries (Hai et al.).

In the mini-review, authors proposed the recent advances in the changes and practical application of garlic products after processing. Initial processing can extend garlic's storage life and improve its marketability. Moreover, garlic undergoes chemical changes during processing, including reduced sulfur compounds and increased bioactive compounds. In this way, the functionality and convenience of garlic products like garlic oil, aged garlic, black garlic, and garlic powder were enhanced. Thus, it is believed that this review is a valuable resource for industry professionals, researchers, and consumers interested in garlic processing effects. As it promotes quality control and consumer education (Sunanta et al.).

Out of the remaining two reviews, one compiles the current state of knowledge about the subject matter offering a valuable resource to researchers, professionals in the field, and industry experts who are interested in utilizing the potential of plant extracts in food formulations. The summary of recent scholarly work establishes a basis for future investigations and advancements in this ever-evolving domain (Plaskova and Mlcek). Lastly, the fourth article, reviewed the efficacy of cryopreservation in upholding the bioactivity and nutritional value of bioflavonoids taking into account factors such as antioxidant capacity and phenolic content. A comprehensive analysis has been provided on the potential consequences of cryopreserved bioflavonoids in the advancement of improved nutraceutical products, with a specific focus on their applications in functional foods and dietary supplements. The presentation encompasses state-of-the-art technologies and methodologies employed shedding light on developments that may pave the way for the preservation and utilization of bioflavonoids. This review contributes to the scientific community's understanding of cryopreservation as a feasible tool for maintaining the bioactivity of bioflavonoids. Furthermore, the findings presented in this article have broader implications for the nutraceutical industry, offering potential methods for enhancing the stability and shelf life of bioflavonoid-rich products (Xiang et al.).

Over and above, adding bioactive ingredients to food products to promote consumer health, protection, and preservation of these extracts from physical hazards, while improving bioactivity is increasingly becoming a trend. Thus, we should invest in the field of green extraction to generate more practical insights to elevate our understanding of this hot topic and help us find better high-quality extracts, and optimized extraction approaches for improving yield, reducing carbon footprint, and increasing shelf life after processing. Moreover, the potential advantages and limitations of cryopreservation and other related techniques such as freeze drying should also be studied.

## Author contributions

AR: Conceptualization, Writing – original draft. AS: Writing – original draft, Writing – review & editing. KK: Writing – original draft, Writing – review & editing.
